# In utero exposure to maternal smoking is associated with DNA methylation alterations and reduced neuronal content in the developing fetal brain

**DOI:** 10.1186/s13072-017-0111-y

**Published:** 2017-01-26

**Authors:** Zac Chatterton, Brigham J. Hartley, Man-Ho Seok, Natalia Mendelev, Sean Chen, Maria Milekic, Gorazd Rosoklija, Aleksandar Stankov, Iskra Trencevsja-Ivanovska, Kristen Brennand, Yongchao Ge, Andrew J. Dwork, Fatemeh Haghighi

**Affiliations:** 1 Friedman Brain Institute, Icahn School of Medicine at Mount Sinai, 1425 Madison Avenue, New York, NY 10029 USA; 2 Department of Neuroscience, Icahn School of Medicine at Mount Sinai, 1425 Madison Ave, Floor 10, Room 10-70D, New York, NY 10029 USA; 3 Department of Psychiatry, Icahn School of Medicine at Mount Sinai, 1425 Madison Avenue, New York, NY 10029 USA; 4 Department of Neurology, Icahn School of Medicine at Mount Sinai, 1425 Madison Avenue, New York, NY 10029 USA; 5 Department of Psychiatry, Columbia University, New York, NY 10032 USA; 6 Department of Pathology and Cell Biology, Columbia University, New York, NY 10032 USA; 7 Medical Epigenetics, James J. Peters VA Medical Center, Bronx, NY 10468 USA; 8 Macedonian Academy of Sciences and Arts, Skopje, Macedonia; 9 School of Medicine, Skopje, Macedonia; 10 Psychiatric Hospital Skopje, Skopje, Macedonia

**Keywords:** Brain, DNA methylation, Epigenetics, Fetal, Neuron, Nicotine, Neurodevelopment, Prenatal, Smoking, Tobacco

## Abstract

**Background:**

Intrauterine exposure to maternal smoking is linked to impaired executive function and behavioral problems in the offspring. Maternal smoking is associated with reduced fetal brain growth and smaller volume of cortical gray matter in childhood, indicating that prenatal exposure to tobacco may impact cortical development and manifest as behavioral problems. Cellular development is mediated by changes in epigenetic modifications such as DNA methylation, which can be affected by exposure to tobacco.

**Results:**

In this study, we sought to ascertain how maternal smoking during pregnancy affects global DNA methylation profiles of the developing dorsolateral prefrontal cortex (DLPFC) during the second trimester of gestation. When DLPFC methylation profiles (assayed via Illumina, HM450) of smoking-exposed and unexposed fetuses were compared, no differentially methylated regions (DMRs) passed the false discovery correction (FDR ≤ 0.05). However, the most significant DMRs were hypomethylated CpG Islands within the promoter regions of *GNA15* and *SDHAP3* of smoking-exposed fetuses. Interestingly, the developmental up-regulation of *SDHAP3* mRNA was delayed in smoking-exposed fetuses. Interaction analysis between gestational age and smoking exposure identified significant DMRs annotated to *SYCE3*, *C21orf56*/*LSS, SPAG1* and *RNU12*/*POLDIP3* that passed FDR. Furthermore, utilizing established methods to estimate cell proportions by DNA methylation, we found that exposed DLPFC samples contained a lower proportion of neurons in samples from fetuses exposed to maternal smoking. We also show through in vitro experiments that nicotine impedes the differentiation of neurons independent of cell death.

**Conclusions:**

We found evidence that intrauterine smoking exposure alters the developmental patterning of DNA methylation and gene expression and is associated with reduced mature neuronal content, effects that are likely driven by nicotine.

**Electronic supplementary material:**

The online version of this article (doi:10.1186/s13072-017-0111-y) contains supplementary material, which is available to authorized users.

## Background

Numerous studies have established that maternal smoking during pregnancy is associated with impaired executive function and behavioral problems in the offspring [1–3]. Maternal smoking is associated with altered fetal brain development [4] and reduced volumes of cortical gray matter in childhood [5], indicating that exposure to tobacco smoke constituents in utero may impact brain development and subsequently result in neurodevelopmental abnormalities. Offspring exposed to smoking after birth does not exhibit the same adverse trajectories [6, 7], suggesting biologically mediated mechanisms during gestation. Cigarette smoke is a highly complex mixture of more than 5000 chemicals of which approximately 100 are known to be hazardous [8]. Linking specific compound(s) with defined phenotypes has proven difficult. Indirect biological mechanisms caused by cigarette constituents other than nicotine have been proposed, including hypoxia/ischemia and DNA damage [9]. Exposure to nicotine prenatally has a direct impact on brain development. In rodents, prenatal exposure to nicotine is reported to induce abnormal dendritic morphology and reduced synapse density in the cerebral cortex and nucleus accumbens [10]. Additionally, prenatal nicotine exposure during primate brain development up-regulates nicotinic acetylcholine receptors (nAChR), causes cell death, and alters cell size and neurite outgrowth in a regionally dependent manner [11]. Furthermore, nicotine replacement therapy has been suggested to increase the risk for behavioral impairments (for review see [12–17]).

Prenatal exposure to environmental factors such as alcohol [18] and industrial chemicals (lead, methylmercury, PCBs, reviewed in [19, 20]) often manifests as neurodevelopmental disorders. Epigenetic modifications such as DNA methylation regulate gene activity necessary for cell differentiation [21]. Exposure to tobacco smoke can induce alterations in epigenetic patterning that are associated with a wide spectrum of human diseases including cardiovascular, pulmonary, neurobehavioral disorders and cancer [22–28]. Maternal smoking during pregnancy alters DNA methylation in the blood of newborns [29] and can cause DNA methylation changes that persist into childhood [30]. In relation to neurological function, differences in DNA methylation have been reported between offspring of smokers and non-smokers in the promoters of catechol-O-methyltransferase (*COMT*) and monoamine oxidase A (*MAOA*), genes thought to be involved in nicotine dependence and other neurobehavioral disorders [31, 32]. Further, an increase in the DNA methylation of the brain-derived neurotrophic factor-6 (*BDNF*-*6*) promoter/5′UTR has been found in adolescents exposed to maternal smoking during pregnancy [33]. To our knowledge, no studies have directly examined the epigenetic changes of the developing human brain exposed in utero to maternal cigarette smoking. Here we interrogate DNA methylation patterns in the developing cortex of human fetuses exposed to maternal smoking on a genome scale.

## Methods

### Sample selection

Fetuses were from second-trimester elective saline abortions performed for non-medical reasons. Fetal sample groups, exposed (*N* = 14) and unexposed (*N* = 10) to maternal smoking, were balanced for sex and gestational age (weeks since the first day of last normal menstrual period) (Table 1). All mothers of the exposed group were active smokers prior to and during pregnancy, whereas no mothers of the unexposed group were active smokers prior to or during pregnancy (Additional file 4: Table S1). Alcohol is a well-described teratogen that affects neuroanatomical development [18, 34]. No mother reported any alcohol abuse or dependence prior to or during pregnancy. A higher proportion of mothers reported consuming some (“any”) alcohol during pregnancy in the unexposed (60%) then exposed (29%) groups, although this difference was not significant (*p* value = 0.12, Chi-Square, Additional file 4: Table S1). All samples were identified as Caucasians.

**Table 1:** Fetal cortical samples dissected from the second trimester (ST) of gestation

	Early ST			Late ST			Total
	*N*	Age in wpc (mean ± SD)	M/F	*N*	Age in wpc (mean ± SD)	M/F	
Exposed	9	16.63 ± 0.52	5/4	5	22.4 ± 1.14	3/2	14
Unexposed	6	16.67 ± 0.52	3/3	4	23.25 ± 0.96	2/2	10

Fetal sample groups, exposed and unexposed to maternal smoking, were balanced for gestational age and sex

### Sample dissection and processing

Upon delivery, the products of conception were refrigerated, and within hours, they were moved to a −80 °C freezer. For examination, they were placed at −20 °C overnight. Working quickly over dry ice, the brain was removed without thawing. The cortical plate was sampled in the region that becomes the DLPFC in order to obtain post-migratory NeuN-immunoreactive (NeuN^+^) neurons, which normally become numerous in cortical layers 4–6 between 14 and 20 weeks gestational age, and in layers 2 and 3 between 20 and 24 weeks (Additional file 1: Figure S1) [35]. This region was chosen because it is involved in decision-making and working memory, and its function is compromised in neurodevelopmental and psychiatric conditions, including autism spectrum disorder (ASD). It is readily identified and accessible in second-trimester fetal brain. During the second trimester, the cerebral hemispheric wall in the frontal region grows from a thickness of ~2 mm at 12 weeks to ~6–8 mm at 18 weeks and ~18 mm at 26 weeks, with cortical plate thickness of ~0.5–1, ~1.5, and ~2 mm, respectively [36–38]. We obtained tissue from the cortical plate from frozen fetal brains by scraping the dorsal prefrontal region of the left hemisphere to a depth of approximately 0.5 mm for the youngest fetuses, where there was no gross demarcation between plate and subplate. In the older fetuses, we were guided by a change in color at the junction of the cortical plate and subplate at approximately the predicted depth. These sample specimens for DNA methylation and gene expression assays were stored at −80 °C for further processing.

### Human induced Neuronal Precursor Cells (hiNPC)

All hiNPC lines were derived as previously described [39]. To match the in vivo data generated from postmortem studies, hiNPC lines (NSB553-3-C, NSB2607-4-1 and NSB690-2-1) used in this study were derived from three Caucasian males, and for full details of the donors of the fibroblasts and validation of the hiPSC and NPC lines, please see [40]. Cell culture; NPCs were maintained at high density, grown on growth factor-reduced Matrigel (BD Biosciences)-coated plates in NPC media (Dulbecco’s Modified Eagle Medium/Ham’s F12 Nutrient Mixture (ThermoFisher Scientific), 1× N2, 1× B27-RA (ThermoFisher Scientific) and 20 ng/ml^−1^ FGF2 and split 1:3 every week with Accutase (Millipore, Billerica, MA, USA). Neural differentiation; NPCs were dissociated with Accutase and plated at 2.0 × 10^5^ cells per cm^2^ in NPC media onto growth factor-reduced Matrigel-coated plates. For neuronal differentiation, medium was changed to neural differentiation medium (DMEM/F12, 1× N2, 1× B27-RA, 20 ng/ml^−1^ BDNF (Peprotech), 20 ng/ml^−1^ GDNF (Peprotech), 1 mM dibutyryl-cyclic AMP (Sigma), 200 nM ascorbic acid (Sigma) 1–2 days later. NPC-derived neurons were differentiated for 3 and 6 weeks before being assayed.

### Nicotine treatment

Nicotine (N0267-100MG, Sigma) was diluted at three different concentrations [100 nM (low), 10 μM (med) and 1 mM (high)] in neuronal media and added every 2nd day with a complete media change. Control wells were treated with equal volume of vehicle (ethanol) added to neuronal media.

### hiNPC assays

#### Toxicity

The cell impermeant nuclei dye TO-PRO3^®^ (ThermoFisher Scientific, T3605) was added at week 3 of differentiation. Three plates, each containing triplicates of a hiNPC line, were imaged with an Odyssey^®^ infrared imaging system (LI-COR). TO-PRO3^®^ fluorescence intensity was normalized to control (vehicle treated) wells.

#### Immunofluorescence analysis

At 3 and 6 weeks of differentiation, cells were washed once with 1× PBS and then fixed in 4% paraformaldehyde (Electron Microscopy Services) for 15 min. Following 3 washes with 1× PBS, cells were then blocked and permeabilized with 1% v/v BSA Fraction V (BSA, ThermoFisher Scientific) with 0.3% v/v Triton-X 100 (T-100X, Sigma). Primary antibodies (Rb-Ki67, 1:500, Abcam, ab15580 and Ms-TUJ1, 1:1000, Covance, MMS-435P) were added overnight in 1%BSA/0.5%T-100X. Appropriate secondary antibodies (AlexaFluor Dk secondaries, Ms-680 and Rb-800) were incubated for 2.5 h in 1%BSA/0.5%T-100X. Following 3 washes with 1× PBS, plates were imaged with an Odyssey^®^ infrared imaging system (LI-COR). Fluorescence intensity was normalized to control wells. Statistical differences between nicotine-treated and vehicle-treated controls were determined by Student’s *t*test using R Language 3.03 [41].

#### Illumina Infinium Human Methylation BeadChip sample processing

DNA from fetal brains and hiNPCs were isolated and bisulfite converted (Zymo Research), and CpG methylation was determined using Illumina Infinium Human Methylation BeadChip microarrays (HM450), as described previously [42].

#### DNA methylation data preprocessing

The analyses were performed using R Language 3.03 [41] an environment for statistical computing and Bioconductor 2.13 [43]. Raw data files (.idat) were processed by minfi package [44]. All samples displayed a mean probe-wise detection call for the 485,512 array probes <0.0005. The data were normalized, background subtracted and further normalized by SWAN [45]. *M* values were used in feature selection models. Beta values (logistic transformed *M* values) were used for sample sex determination and DNA methylation reporting. Probes mapping to multiple locations (*N* = 19,834), Infinium type I probes with a SNP at the interrogated CpG (*N* = 13,708) and probes mapping to the X- and Y-chromosomes were removed from analysis (*N* = 11,648), as described [46], leaving 452,930 analyzable probes.

#### DNA methylation analysis

Differentially methylated probes (DMPs) display a mean difference in DNA methylation of at least 20%, corresponding to a methylation difference detectable by the HM450 with 99% confidence [47]. DMPs were mapped to refSeq gene annotations and analyzed using Ingenuity Pathways Analysis (IPA) software (Ingenuity Systems, www.ingenuity.com). Differentially methylated regions (DMR) were found using the bumphunter algorithm applied to DNA methylation *M* values [48]. Specifically, for each CpG site, we estimate the difference between the *M* values for the exposed and unexposed adjusting for gestational age, sex and sample chip assignment. An interaction term was included between smoking exposure and gestational age for interaction DMR analysis. The methylation difference estimates are smoothed based on the predefined CpG clusters where the maximal gap between neighboring CpG sites is 500 bp, while the largest cluster size is set to 1500 bp. The smoothed regional methylation difference estimates were obtained using a predefined threshold to identify the putative DMRs, with associated significance levels obtained empirically based on 1000 permutations. Cell-proportion estimates were performed using the methods described in Jaffe et al. [49]. Briefly, publicly available HM450 data from ESC-derived NPC (H9) [50], adult cortical NeuN^+^ and NeuN^−^ cells [51] were quantile normalized together [44] and 227 unique probes that separated the 3 cell types were used in a nonlinear mixed modeling [52] to estimate the proportion of each of the 3 cell types within our HM450 fetal dataset. Cell-proportion estimates were also generated for publicly available HM450 data from dissected postnatal DLPFC aged 4, 6 and 10 months, produced by the BrainSpan Consortium [53].

#### Gene expression analysis

Total RNA was isolated from the same 24 fetal samples used for DNA methylation analysis (ToTALLY RNA™ Total RNA Isolation Kit, Ambion). Fetal mRNA was analyzed using Nanostring nCounter Elements technology. Gene expression analysis of fetal DLPFC samples was performed for the 2 most significant smoking-DMRs (SDHAP3 and GNA15) and 3 of the 5 genes annotated to the most significant interaction DMRs (C21orf56, POLDIP3 and SYCE3). Housekeeping gene selection: We used the Nanostring nCounter Elements technology and selected 4 housekeeping genes for expression normalization. Previously, Penna et al. [54] investigated the stability of a panel of housekeeping genes for mRNA normalization in human postmortem brain samples. Additionally, Madden et al. [55] described a subset of ubiquitously expressed transcripts ideal for using as housekeeping genes within brain tissue. We selected 4 housekeeping genes, 3 of which were identified by both Penna et al. and Madden et al. (*GAPDH, YWHAZ* and *CYC*) and *SDHA*, identified by the former group and that we have previously used successfully in Nanostring interrogation of rat mRNA [56]. Negative control subtraction and normalization to housekeeping genes was performed using the nSolver Analysis Software. Sample fold-changes (FC) were calculated relative to sample FS5777 (one of the 24 fetal samples analyzed chosen at random) gene expression levels for each gene independently. Any expression values of 3 standard deviations from the group mean were deemed outliers and removed from the analysis. No more than 1 result for any assay was removed.

## Results

### Cortical sampling

Fetal samples exposed to maternal smoking were matched to unexposed fetal samples by age and sex when available (Table 1). Fetal brain weight and total weight were highly correlated (*R* ^2^ = 0.94, Additional file 1: Figure S1a), and within either early or late second-trimester samples, no significant difference in brain weight was observed between exposure groups (*p* value = 0.3 and 0.8, respectively, Student’s *t* test) (Additional file 1: Figure S1b, c). The cortical plate was sampled from the presumptive DLPFC in an effort to obtain post-migratory NeuN^+^ neurons, which normally become numerous in cortical layers 4–6 between 14 and 20 weeks gestational age, and in layers 2 and 3 between 20 and 24 weeks [57].

### Maternal smoking-associated differential DNA methylation in the fetal cortex

**Fig. 1:**
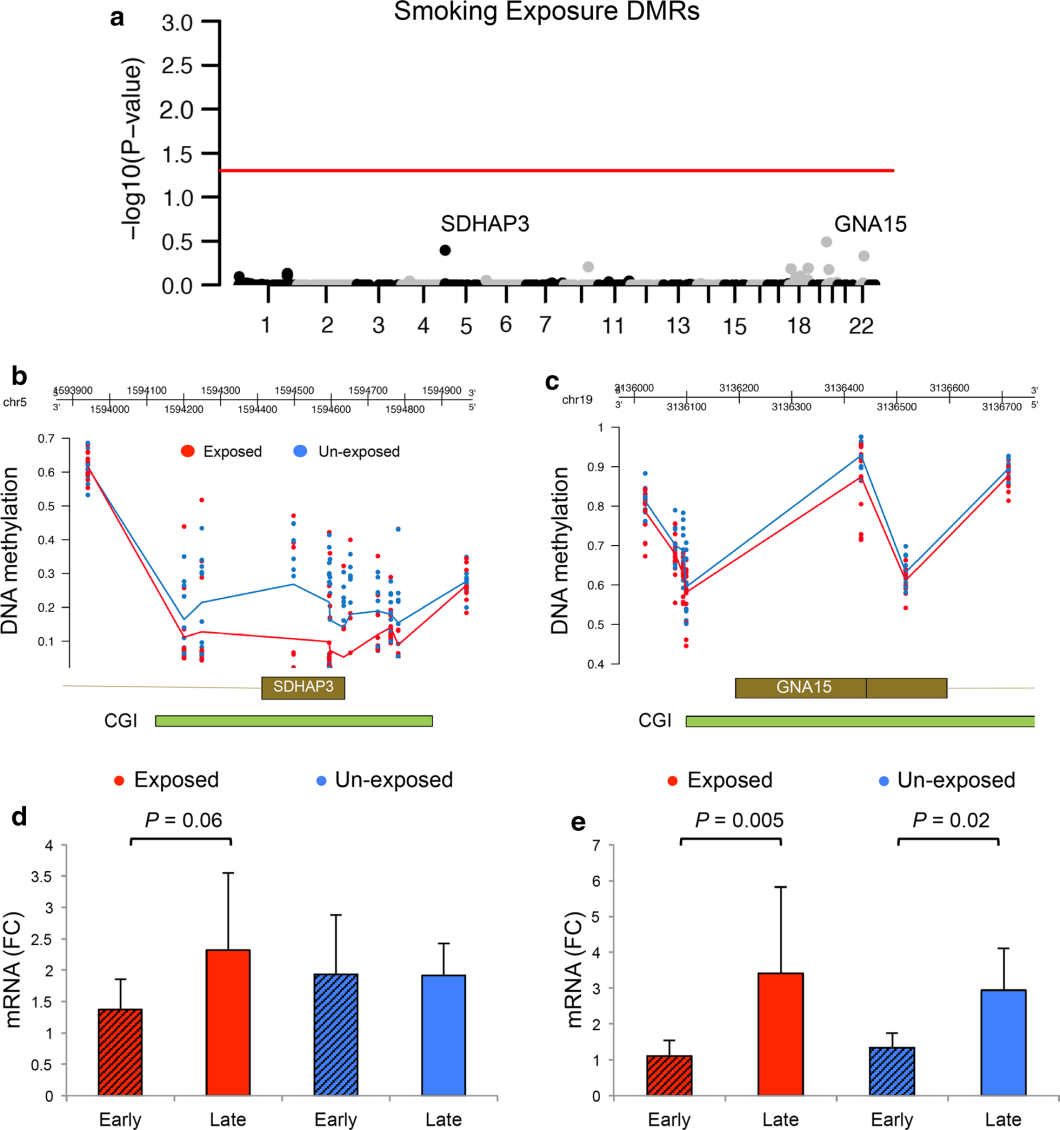
Differentially methylated regions associated with fetal smoking exposure. **a** Manhattan plot shows the most significant smoking exposure DMRs identified between smoking-exposed and unexposed fetal cortical samples, *red line*; adjusted *p* value = 0.05. DNA hypomethylation of fetal cortical samples exposed to maternal smoking was found within *the promoter regions of* the two most significant smoking exposure DMRs **b** *SDHAP3* and **c** *GNA15*. *CGI* CpG Island. Gene expression of **d** *SDHAP3* and **e** *GNA15 shows temporal up*-*regulation in the fetal cortex, particularly in smoking exposed*

To examine DMRs associated with maternal smoking exposure in the fetal cortex, we performed DNA methylation microarray analysis among exposure groups (“Methods” section). Across the 452930 CpG sites examined, no DMRs passed multiple testing correction (family wise error rate, fwer, cutoff ≤0.05, Fig. 1a). This was likely due to the small number of fetal brains available for analysis. Notably, smoking exposure DMRs with the highest point-wise significance was found within the gene promoters of S*DHAP3* and G protein subunit Alpha 15 (*GNA15*) (Fig. 1b, c). Both DMRs were hypomethylated in smoking exposed (Fig. 1b, c). Gene expression analysis of SDHAP3 and *GNA15* (Fig. 1d, e) did not reveal exposure-related differences. However, we observed up-regulation of mRNA between early and late second-trimester samples that were restricted to smoking-exposed fetuses for expression of *SDHAP3* and larger for exposed than for unexposed for expression of *GNA15* (*p* value = 0.005 vs 0.02, Fig. 1e). These observations led us to hypothesize that maternal smoking exposure has temporal effects on gene expression and DNA methylation during fetal cortical development.

### Developmental interaction with maternal smoking exposure

**Fig. 2:**
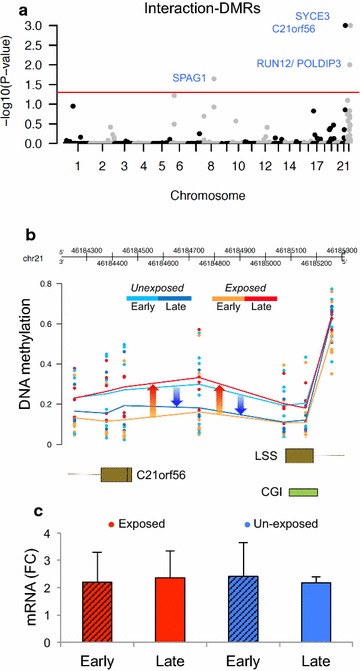
Differentially methylated regions associated with gestational age and maternal smoking exposure interaction (interaction DMRs). **a** Manhattan plot shows the most significant interaction DMRs. DMRs < adjusted *p* value 0.05 are highlighted in *blue. Red line*; adjusted *p* value = 0.05. **b** Example of *an interaction DMR* spanning a bidirectional promoter of *C21orf56* and *LSS* exhibits DNA hypermethylation between early second trimester (ST) and late ST from smoking-exposed fetal cortex samples, whereas unexposed fetal cortex samples exhibit hypomethylation. *CGI;* CpG Island **c** Gene expression (mRNA fold change) of *C21orf56* shows no difference in fetal cortex by smoking exposure or stage of gestation

To explore the effects of smoking exposure on fetal development, we performed an interaction DMR analysis between gestational age and smoking exposure (“Methods” section). Four significant DMRs (fwer *p* value ≤ 0.05) located within the promoters of synaptonemal complex central element protein 3 (*SYCE3*), chromosome 21 open reading frame 56 (*C21orf56*/*LSS*), sperm-associated antigen 1 (*SPAG1*) and *RNA,* U12 Small Nuclear (*RNU12*/*POLDIP3*) (Fig. 2a) were identified. Within the promoter region of *C21orf56*, exposed fetal cortices at 22.4 weeks (late) exhibited higher total DNA methylation levels than samples examined at 16.6 weeks (early). Conversely, unexposed fetal cortices displayed higher DNA methylation levels in early samples compared to late (Fig. 2b). Additionally, we found an intragenic region of *SPAG1* that was conservatively hypomethylated in all samples except a subset of late second-trimester exposed fetal cortices that were hypermethylated (Additional file 2: Figure S2). Nanostring expression analysis of *C21orf56* demonstrated no difference in mRNA levels between exposure/developmental groups (Fig. 2c).

No discernable difference in group-wise DNA methylation patterns by exposure/development was evident in the promoter regions of *RUN12*/*POLDIP3* or *SYCE3*, regions that had been identified using the bumphunter algorithm as significant (Additional file 3: Figure S3a, b). Gene expression analysis of *SYCE3* revealed low gene expression in early second-trimester smoking exposed (*p* value = 0.02) (Additional file 3: Figure S3c), analogous to the *SDHAP3* mRNA results indicative of developmental delay in smoking exposed. No difference in *POLDIP3* mRNA was found between exposure/development (Additional file 3: Figure S3d).

### Global DNA methylation of fetal cortex exposed to maternal smoking

**Fig. 3:**
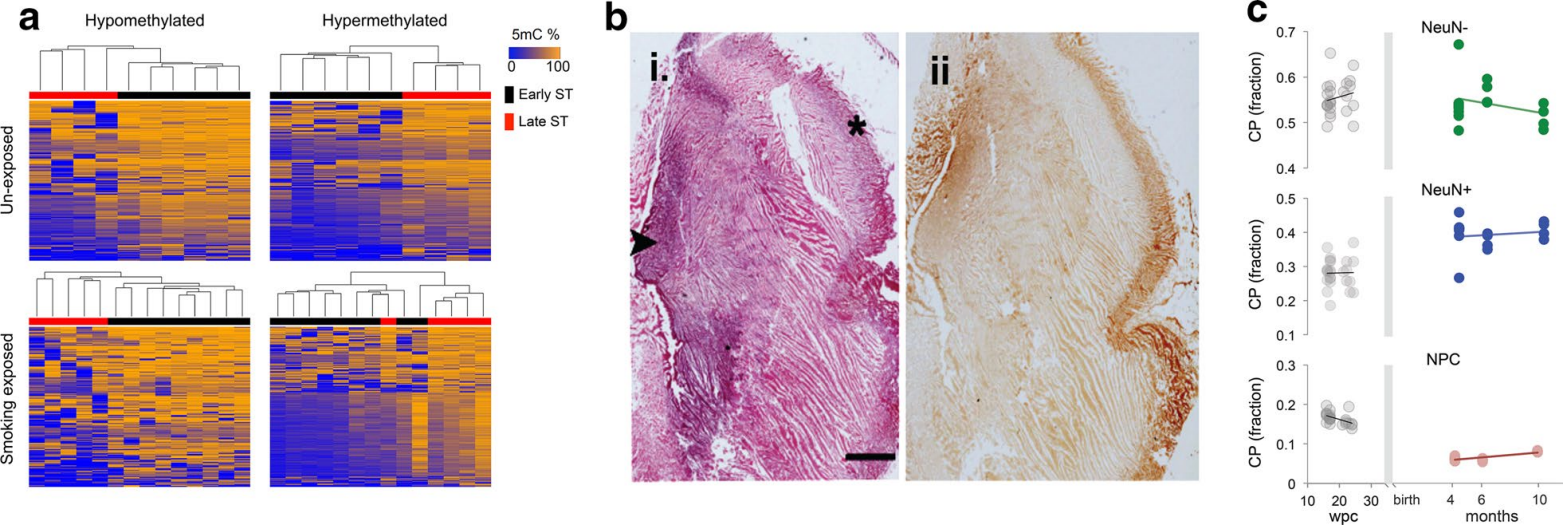
DNA methylation patterns of the developing fetal DLPFC. **a** Heatmaps of unsupervised hierarchical clustered DMPs found differentially methylated between early and late ST for each exposed and unexposed group. **b** Coronal sections from formalin-fixed, paraffin-embedded, previously frozen fetal cortex (20 wpc). Frontal region of cerebral hemisphere showing (*i*) hematoxylin and eosin (H&E) staining and (*ii*) NeuN staining, which distinctly labels the cortical plate (asterisk on H&E) and germinal matrix (*arrowhead*) (Scale = 2 mm). **c** CP estimates of NeuN^−^, NeuN^+^ and NPC within exposed and unexposed (local) fetal DLPFC samples (*gray*) were compared to CP estimates of publicly available postnatal frontal cortex generated by BrainSpan consortium (*colored*) (“Methods” section)

We examined global DNA methylation changes during cortical development in response to maternal smoking by calculating differentially methylated probes (DMPs) between early and late second-trimester samples in each exposure group (methods). We identified 574/371 hyper/hypomethylated DMPs (Additional file 4: Table S2) in unexposed samples and 399/178 hyper/hypomethylated DMPs (Additional file 4: Table S3) in exposed samples (Fig. 3a). Unsupervised hierarchical clustering of both hypomethylated and hypermethylated DMPs separated samples by gestational age (Fig. 3a). More DMPs were found between early and late second-trimester unexposed samples than between smoking-exposed samples. Notably, a higher proportion of these DMPs were hypermethylated in the unexposed (64%) compared to exposed samples (44%). Hypomethylation is a feature of pluripotent/multipotent stem cells and as cells differentiate, the acquisition of DNA methylation restricts cell lineage and drives cell specification [58]. We postulated that reduced hypermethylated DMPs in smoking-exposed samples could reflect alterations or delay in cell-type specification occurring in the developing cortices in response to smoking exposure. Gene ontology analysis implicated canonical pathways of “Role of NFAT in Cardiac Hypertrophy,” “Th2 Pathway” and “Th1 and Th2 Activation Pathway” associated with genes annotated to DMPs between early and late second-trimester unexposed fetal samples (Additional file 4: Table S4). NFAT is a transcription factor that mediates axon growth in developing neurons (reviewed in [59]). Conversely, canonical pathways associated with genes annotated to DMPs between early and late second-trimester exposed fetal samples implicated “T Helper Cell Differentiation,” “Epithelial Adherens Junction Signaling” and “Factors Promoting Cardiogenesis in Vertebrates” (Additional file 4: Table S4).

Our cortical plate sectioning of the presumptive DLPFC aimed to enrich for post-migratory NeuN^+^ neurons (Fig. 3b). Other investigators have established cell deconvolution algorithms with remarkable accuracy in estimating NeuN^+^ proportions of whole brain tissues using DNA methylation profiles [51, 60]. Using the DNA methylation profiles generated from the fetal cortices, we were able to estimate the cell proportions (CP estimates) of NeuN^+^, NeuN^−^ and neural precursor cells (NPCs) within the fetal DLPFC sections (“Methods” section). CP estimates revealed our fetal cortical sections contained a high proportion of NPCs (mean = 16%) compared to CP estimates of NPCs of cortical sections (postnatal 4–10 months) produced by the BrainSpan consortium (mean = 7%) [50, 53, 57] (Fig. 3c). These NPCs are presumably undergoing maturation, and thus, our sections provide a rare window into the effects of maternal smoking exposure on neuro-cellular development.

**Fig. 4:**
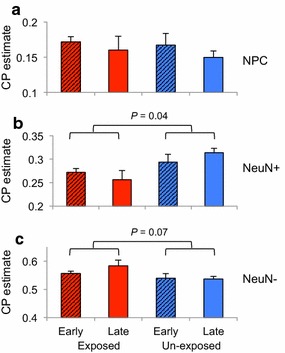
Cell proportion (CP) estimates by exposure and gestational age in the developing DLPFC. *Bar plots* show DNA methylation-based CP estimates of NPCs, NeuN^+^ and NeuN^−^ (**a**–**c**, respectively) within fetal samples split by smoking exposure and gestational age. Significantly fewer (proportionally) NeuN^+^ were observed between smoking exposed (all samples) and unexposed (all samples)

CP estimates of fetal DLPFC revealed significantly fewer NeuN^+^ in smoking-exposed fetal DLPFC (*p* value = 0.04, Fig. 4). Any decrease in CP estimate will be balanced by an increase in the proportion of another cellular population, and indeed, we observed a higher proportion of NeuN^−^ in smoking-exposed fetal samples (*p* value = 0.08, Fig. 4). No difference in NPC proportions was observed between smoking exposure groups (Fig. 4). The results indicate that tobacco exposure is associated with a reduction in NeuN^+^ in the developing DLPFC. Using ReFACTor [61] method that adjusts for the possible effect of variation in cell-type proportions, we found no difference in methylation associated with smoking exposure or developmental stage interaction. The methylation change associated with smoking exposure reflects differences in rate of cell differentiation during development as observed by the estimated CP changes. To investigate the mechanism of nicotine exposure on neuron development, we turned to an in vitro model of development.

### In vitro modeling of neurodevelopment in response to nicotine exposure

**Fig. 5:**
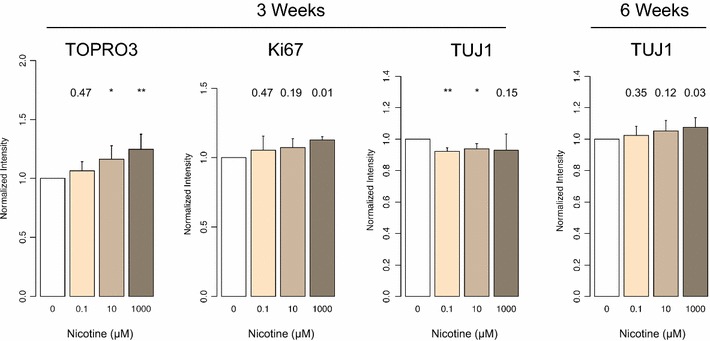
Immunohistochemistry (IHC) staining on hiNPCs. *Left to right* significant changes in IHC staining of hiNPCs at week 3 for *TOPRO3, Ki67* and *TUJ1* and at 6 weeks for TUJ1. Significance of difference compared to vehicle-treated control (*p* value, Student’s *t* test) specified above each result; * *p* value <0.01; ** *p* value <0.001

To model the effects of nicotine on human neuronal development, we exposed human induced neural precursor cells (hiNPCs) to nicotine over a 6-week period of neuronal differentiation. Following 3 weeks of nicotine exposure, differentiating hiNPCs exhibited a dosage-dependent increase in TO-PRO^®^-3 fluorescence (live cells are impermeable to the dye, whereas the dye penetrates compromised membranes characteristic of dead cells) (*p* value <0.05) and an increase in cell proliferation (*Ki67*) that was significant at high dose (*p* value = 0.01, Fig. 5). We observed a decrease in the staining of βIII Tubulin (*TUJ1*), a marker for mature neurons, and notably, the most significant decrease was found at 0.1 uM (low) nicotine exposure (*p* value = 0.0003, Fig. 5). No significant differences in cell death or proliferation were detectable by 6 weeks; however, *TUJ1* showed a dosage-dependent increase that was significant at 1000 μM nicotine (*p* value = 0.03, Fig. 5). These data demonstrate that nicotine elicits dosage-dependent effects on neuronal maturation. Interestingly, the biologically meaningful nicotine exposure (0.1 μM) restricts early neuron development despite exhibiting a lower toxicity (cell death) than higher exposures. Further, the differences in *TUJ1* staining between 3 and 6 weeks indicate that less differentiated hiNPCs (3 weeks) are more susceptible to the inhibiting effects of nicotine on neuron development (Fig. 5).

## Discussion

In this study, we profiled genome-scale DNA methylation of fetal brain development in response to exposure to maternal smoking in utero. Although limited in number, the fetal brain samples have well-characterized maternal health history and exposure data, thus providing a rare opportunity to investigate the impact of nicotine exposure on early human brain development. Notably, our DNA methylation profiling was performed on whole tissue sections from the developing DLPFC that consist of a mixture of neuronal and non-neuronal cell types. Established methods enable the isolation of neuronal nuclei [62]; however, due to the fragility of fetal neuronal nuclei, this technique cannot be applied. Estimating cell proportions using DNA methylation profiles revealed our fetal DLPFC sections contained approximately 16% NPCs that are presumably undergoing maturation, providing a rare window into human neuro-cellular development in response to maternal smoking exposure.

Embryogenesis is a stage of rapid neurological transformation and growth in which epigenomic landscapes undergo dramatic change [63, 64]. In the developing DLPFC, genome-wide DNA methylation changes that distinguish early and late second-trimester samples were clearly reduced in fetal cortex of smoking exposed indicating alterations in cell-type differentiation. Indeed, our most significant DMRs were identified by interaction analysis between gestational age and maternal smoking exposure. We found a delay in the up-regulation of expression *of SYCE3*, a gene that is conserved among mammals and whose loss leads to a block in synapsis initiation resulting in meiotic arrest [65]. We also identified an interaction DMR in the promoter of *C21orf56* (also known as *SPATC1L*). *C21orf56* is a spermatogenesis and centriole-associated 1-like gene found on chromosome 21 of little-known function. Although we did not observe developmental/exposure dependent changes in gene expression, we observed a gain in promoter methylation in late second-trimester exposed samples that could reduce the transcriptional potential of *C21orf56* later in development.

Genes such as *GNA15* and *SDHAP3* that contained maternal smoking-associated DMRs displayed a developmental delay in mRNA up-regulation in smoking exposed. Notably, *SDHAP3* is a subunit of the succinate dehydrogenase complex located within the mitochondrial membrane and functions in electron transport chain transfer of electrons to coenzyme Q [66]. It has been reported that mutations within succinate dehydrogenase subunits actually increase levels of oxidative stress [67]. Intriguingly, this same DMR was recently found hypermethylated in the cerebellum of patients diagnosed with ASD [68] and differentially methylated in the DLPFC of patients diagnosed with schizophrenia (SCZ) within 3 independent studies [69]. Furthermore, in a separate report, *GNA15* was found to be differentially methylated in the PFC of ASD patients [70]. Both ASD and SCZ probably have prenatal origins [71–73]. Taken together, these results reveal a potential link between maternal smoking-associated DNA methylation perturbation and potential increase risk for neurodevelopmental abnormalities. Notably, *GNA15* is transcriptionally modifiable by acute doses of nicotine in neuroblastoma cell lines [74], indicating nicotine as a potential causative agent.

Cell deconvolution algorithms have shown remarkable accuracy in estimating NeuN^+^ proportions from DNA methylation profiles from whole brain tissue [51, 60]. CP estimates within our fetal DLPFC revealed a smoking exposure-associated reduction in NeuN^+^ cells supporting previous observations of reduced gray matter in the cortex of smoking-exposed children [5]. The adverse effects of maternal smoking on fetal development are well described; however, it was estimated that 30% of smokers attempting to quit smoking use cessation aids that contain nicotine [75]. Nicotine is a well-studied substance in tobacco and has been shown to induce oxidative stress in rodent [76, 77] and human neurons [78]. Exposure of hiNPCs to 100 nM nicotine resulted in the lowest amount of toxicity but the greatest suppression of neuronal differentiation (B3-Tubulin). These results recapitulate the reduction in the estimated proportion of NeuN^+^ cells we observed in human samples and implicate nicotine as a causative agent *in* impeding neuronal development. These data provide direct evidence from primary tissue of in utero exposure to teratogenic agents as found in cigarettes—warranting further investigations of the in utero environment on fetal development and how it impacts offspring health and disease risk through the lifespan.

## Conclusions

In summary, we have found evidence that intrauterine smoking exposure alters the developmental patterning of DNA methylation and gene expression and is associated with reduced mature neuronal content, effects that are likely driven by nicotine through mechanisms independent of cell death.

## Additional files

**Additional file 1**: **Fig.** **S1**. Fetal brain weight and total body weight of fetus. **a** Correlation between fetal brain weight and total fetal body weight. The change in total body weight (**b**) and brain weight (**c**) in exposed and unexposed fetal samples between early ST and late ST. * *p* value <0.05, ** *p* value >0.1. Error bars = SD.

**Additional file 2**: **Fig.** **S2**. DNA methylation of fetal samples by exposure and gestational age of the significant intergenic interaction DMR found within *SPAG1.* CGI; CpG Island.

**Additional file 3**: **Fig.** **S3**. DNA methylation of fetal DLPFC by exposure and gestational age within interaction DMRs annotated to the promoters of **a** *SYCE3* and **b** *POLDIP3*/*RNU12*. *CGI* CpG Island. *Gene expression* analysis revealed temporal up-regulation of c *SYCE3* in smoking *exposed*; however, gene expression analysis of **d** *POLDIP3*/*RNU12* shows no significant difference between smoking-exposed or unexposed early ST and late ST.

**Additional file 4**: **Table** **S1**. Results of mothers self-reported family history of environmental exposures prior and during pregnancy. **Table** **S2**. DMPs (late–early) smoking unexposed. **Table** **S3**. DMPs (late–early) smoking exposed. **Table** **S4**. Top (significance) canonical pathways associated with genes annotated to DMPs found between late and early ST within smoking-exposed and unexposed fetal samples.

## Abbreviations

ASD: autism spectrum disorder
BDNF-6: brain-derived neurotrophic factor-6
C21orf56: chromosome 21 open reading frame 56
COMT: catechol-O-methyltransferase
CP: cell proportion
DLPFC: dorsolateral prefrontal cortex
DMP: differentially methylated positions
DMR: differentially methylated region
FDR: false discovery rate
*GNA15*: G protein subunit alpha 15
hiPSCs: human induced pluripotent stem cell
HM450: Illumina Infinium Human Methylation Microarray 450K platform
IHC: immunohistochemistry
MAOA: monoamine oxidase A
nAChR: nicotinic acetylcholine receptors
NeuN^+^: NeuN-immunoreactive
NPC: neural progenitor cell
RNU12: RNA, U12 Small Nuclear
SCZ: schizophrenia
SPAG1: sperm-associated antigen 1
CGI: CpG Island
