# Supplementary figures and images for: In utero exposure to maternal smoking is associated with DNA methylation alterations and reduced neuronal content in the developing fetal brain
Source: Epigenetics Chromatin. 2017 Jan 26;10:4. doi: 10.1186/s13072-017-0111-y (PMC5270321; doi:10.1186/s13072-017-0111-y)

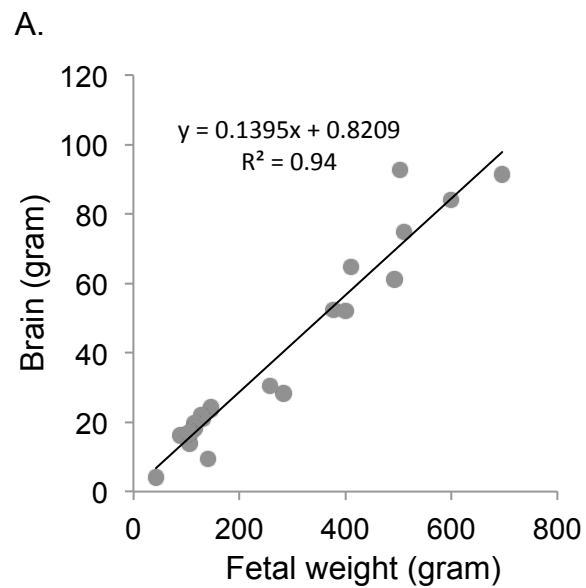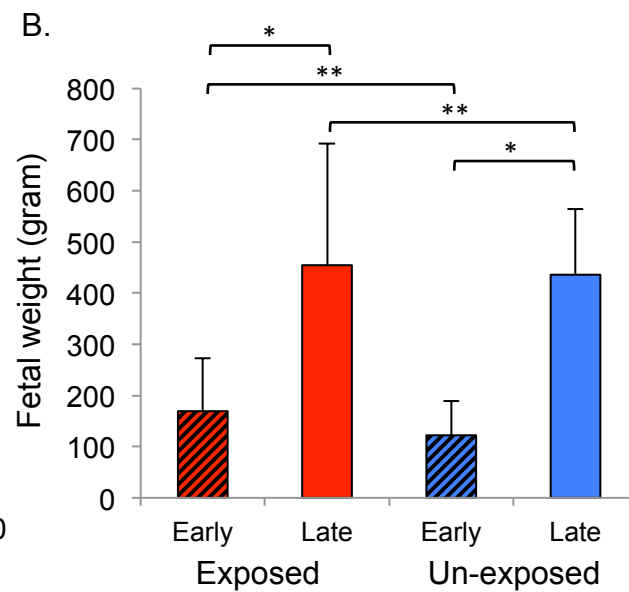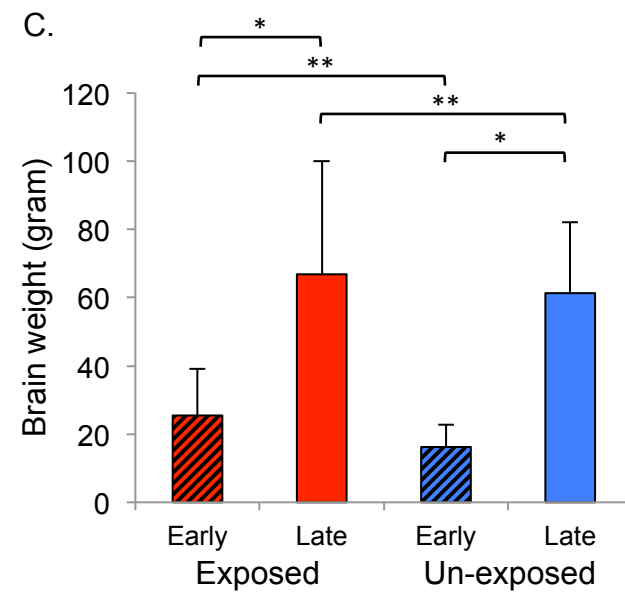

Supplementary Figure 1.

Supplement: Supplementary file 1 — Additional file 1: Fig. S1. Fetal brain weight and total body weight of fetus. a Correlation between fetal brain weight and total fetal body weight. The change in total body weight (b) and brain weight (c) in exposed and unexposed fetal samples between early ST and late ST. * p value <0.05, ** p value >0.1. Error bars = SD. [file 13072_2017_111_MOESM1_ESM.pdf]

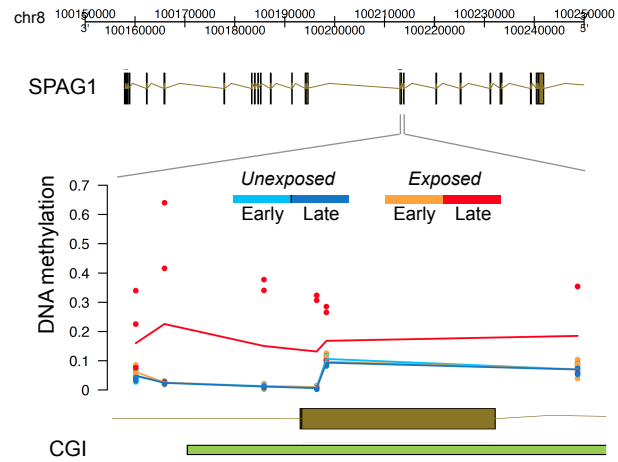

Supplementary Figure 2.

Supplement: Supplementary file 2 — Additional file 2: Fig. S2. DNA methylation of fetal samples by exposure and gestational age of the significant intergenic interaction DMR found within SPAG1. CGI; CpG Island. [file 13072_2017_111_MOESM2_ESM.pdf]

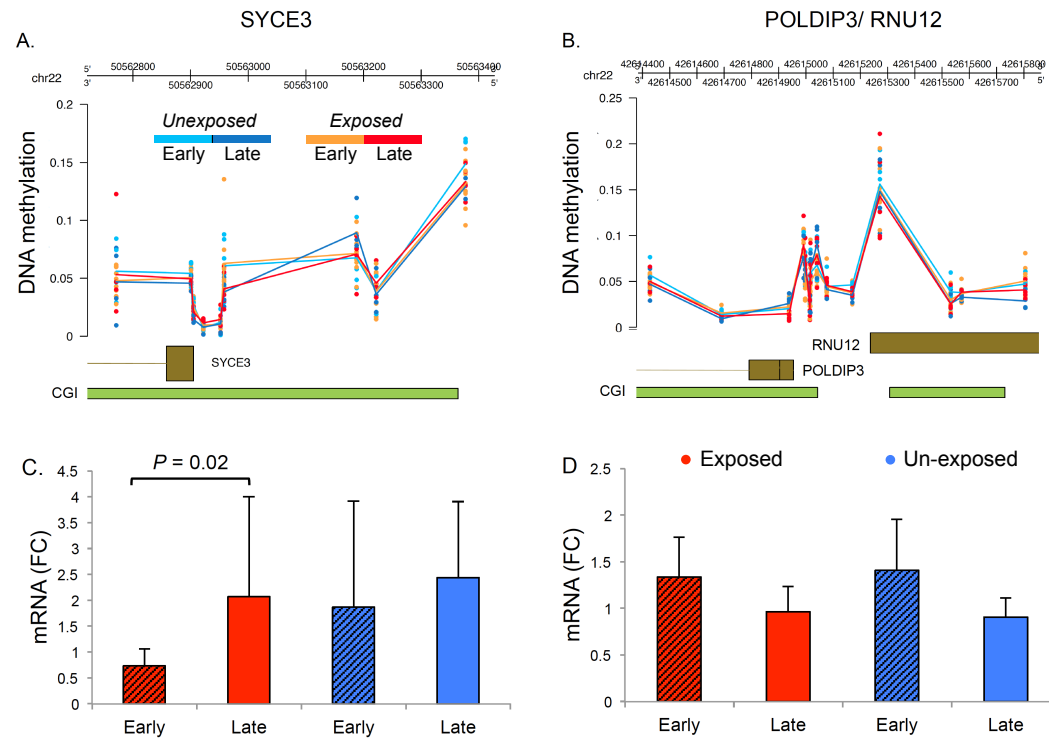

Supplementary Figure 3.

Supplement: Supplementary file 3 — Additional file 3: Fig. S3. DNA methylation of fetal DLPFC by exposure and gestational age within interaction DMRs annotated to the promoters of a SYCE3 and b POLDIP3/RNU12. CGI CpG Island. Gene expression analysis revealed temporal up-regulation of c SYCE3 in smoking exposed; however, gene expression analysis of d POLDIP3/RNU12 shows no significant difference between smoking-exposed or unexposed early ST and late ST. [file 13072_2017_111_MOESM3_ESM.pdf]
